# Conditional cash transfers, uptake of maternal and child health services, and health outcomes in western rural China

**DOI:** 10.1186/s12889-020-08996-9

**Published:** 2020-06-05

**Authors:** Huan Zhou, Yuju Wu, Chengfang Liu, Chang Sun, Yaojiang Shi, Linxiu Zhang, Alexis Medina, Scott Rozelle

**Affiliations:** 1 West China School of Public Health and West China Forth Hospital, Sichuan University, Chengdu, China; 2 China Center for Agricultural Policy, School of Advanced Agricultural Sciences, Peking University, Beijing, China; 3 Center for Experimental Economics in Education, Shaanxi Normal University, Xi’an, China; 4 Center for Chinese Agricultural Policy, Institute of Geographical Sciences and Natural Resource Research, Chinese Academy of Sciences, Beijing, China; 5 Freeman Spogli Institute for International Studies, Stanford University, Palo Alto, California USA

**Keywords:** Maternal and child health, Conditional cash transfer, Impact evaluation, Western rural China

## Abstract

**Background:**

Empirical evidence suggests that the uptake of maternal and child health (MCH) services is still low in poor rural areas of China. There is concern that this low uptake may detrimentally affect child health outcomes. Previous studies have not yet identified the exact nature of the impact that a conditional cash transfer (CCT) has on the uptake of MCH services and, ultimately, on child health outcomes. The objective of this study is to examine the relationship between CCT, uptake of MCH services, and health outcomes among children in poor rural areas of western China.

**Methods:**

We designated two different sets of villages and households that were used as comparisons against which outcomes of the treated households could be assessed. In 2014, we conducted a large-scale survey of 1522 households in 75 villages (including 25 treatment and 50 comparison) from nine nationally designated poverty counties in two provinces of China. In each village, 21 households were selected based on their eligibility status for the CCT program. Difference-in-difference analyses were used to assess the impact of CCT on outcomes in terms of both intention-to-treat (ITT) and average-treatment-effects-on-the-treated (ATT).

**Results:**

Overall, the uptake of MCH services in the sample households were low, especially in terms of postpartum care visits, early breastfeeding, exclusive breastfeeding, and physical examination of the baby. The uptake of the seven types of MCH services in the CCT treatment villages were significantly higher than that in the comparison villages. The results from both the ITT and ATT analyses showed that the CCT program had a positive, although small, impact on the uptake of MCH services and the knowledge of mothers of MCH health issues. Nonetheless, the CCT program had no noticeable effect on child health outcomes.

**Conclusions:**

The CCT program generated modest improvements in the uptake of MCH services and mothers’ knowledge of MCH services in poor rural areas of Western China. These improvements, however, did not translate into substantial improvements in child health outcomes for two potential reasons: poor CCT implementation and the low quality of rural health facilities.

## Background

Improving maternal and child health (MCH) is one of the targets of the United Nation’s Sustainable Development Goals [1]. The Chinese government has made great progress in improving MCH across large parts of the country in recent years by aggressively expanding the coverage of rural health insurance and promoting maternal delivery in hospitals [2–6]. Nevertheless, there are concerns about the status of MCH in Western China’s poor rural areas. According to the literature, up to 40% of women do not receive prenatal physical examinations, and the rate of maternal delivery in hospitals was approximately only 30% in 2012 [7]. In 2014, the maternal mortality ratio was 23.6 per 100,000 live births, which is more than twice the ratio found in eastern regions of China during the same period [8]. When compared with eastern and central regions of China, these western regions also had the highest under-5 mortality rates and neonatal mortality rates in 2015—an estimated under-5 mortality rate of 18.5 deaths per 1000 live births and neonatal mortality rate of 9.5 per 1000 live births [9].

Although one reason for the low uptake of MCH services in these areas may be supply-side challenges, such as poor health service quality and the attitudes of doctors [10, 11], a less-studied aspect of the continuing challenge of promoting higher uptake of MCH services is demand-side factors. The limited research that has been published on this topic shows that the level of a woman’s education, household annual income, and cost of hospital delivery and transportation to the hospital are important factors that correlate with the uptake of prenatal physical examinations and hospital deliveries in rural areas [12–14]. As such, demand-side factors seem to be an important source of differences in MCH service uptake among subpopulations, possibly even more important than supply-side factors [15].

Conditional cash transfer (CCT) programs are an increasingly popular method of improving participation in education and health services in high-, middle-, and low-income countries [16–18]. In their most basic form, CCT programs seek to overcome demand-side constraints by providing cash payments to poor households for behaving in a certain predefined and socially responsible way [19]. In recent years, a number of systematic reviews found that CCTs had positive effects on MCH through addressing health-related social determinants. The CCT program can increase household income and the ability for a household to pay for health services, reduce the cost of care-seeking for using MCH health services (via subsidies), and increase access to MCH services, resulting in improved MCH health [20–22]. There are, however, cases in the literature in which CCT programs did not work [23, 24]. Given that CCT programs have both succeeded and failed in other middle-income countries, we are interested in whether a CCT will succeed or fail in the context of poor rural China.

Previous research conducted within China suggests that CCT programs may be effective in improving MCH uptake in rural areas in China. As stated above, CCT provides cash payments as a way to incentivize certain socially responsible behaviors. From our previous studies, it seems that this cash incentive should be highly effective; when “women’s representatives,” officials charged with managing MCH in villages, were asked about the biggest obstacle to the increased uptake of MCH services by rural women in our sample areas, the most common answer was that costs were too high. In addition, all surveyed village women’s representatives believed that paying women to uptake MCH services—most likely through a CCT program—would be an effective way to increase uptake [15]. A CCT program could, therefore, be an effective way to address these demand-side issues, as these cash transfers would not only resolve the main obstacle that prevents women from using MCH services but also may provide a strong, positive incentive for women to utilize MCH services. We therefore suspect that a CCT program has the potential to be very successful in rural China.

CCTs (conducted by researchers) have been previously used to reduce dropout rates among poor junior high school students in China, and this CCT program was found to significantly reduce dropout by 60% in these areas [25]. In the field of public health, CCTs have been found to have significant effects on the nutritional knowledge and feeding practices of caregivers in rural China [26]. Further, research conducted in the same areas found that providing economic assistance may be an important means to modestly improve the uptake of MCH services [15].

With the goal of improving the uptake of MCH services in poor rural areas, China’s government, with support from the United Nations International Children’s Emergency Fund (UNICEF), launched a CCT pilot program in 2013 in which pregnant women and mothers with infants were incentivized with cash payments to utilize government-provided MCH services. To the best of our knowledge, this program is the first CCT initiative in the health field in China, and, thus, there has not yet been a rigorous impact evaluation of the effectiveness of a CCT program in China’s health field. Our overall goal is to measure the effects of China’s first CCT program on three sets of outcomes: MCH service utilization, mother’s knowledge of MCH issues, and child health outcomes among the poor western rural population in China.

## Methods

### The MCH CCT program

The MCH CCT project office, supported by the government of China, launched the MCH CCT pilot program in late spring 2013 in 40 townships (CCT townships) in three provinces: 11 in Gansu, 17 in Sichuan, and 14 in Yunnan. All villages within each CCT township were offered CCT services. It is important to note, however, that not all townships in these three provinces were CCT townships. The purpose of the CCT program was to encourage eligible pregnant women and mothers to use MCH services. This was expected to lead to better knowledge of MCH and better child health outcomes. All households from targeted towns with pregnant women or neonates during the implementation of the CCT program were eligible.

The premise of the CCT intervention is simple. In a series of group meetings between eligible participants in a selected number of project villages in designated project townships, pregnant women were advised that, if they undertook any one of a set of seven MCH services—of which five are free—then they would receive not only the benefits of the service that was provided but also a cash payment of a certain amount. The program was designed to make payments to the participants shortly after completion of each visit to the MCH service provider.

The list of services and the payment schedule were displayed prominently in all treatment townships. Eligible women were told that they would receive a separate payment each time they (a) underwent a prenatal examination; (b) delivered their baby in a hospital; (c) underwent a maternal postpartum examination; (d) engaged in early breastfeeding, that is, began breastfeeding within one hour after delivery; (e) breastfed exclusively for 6 months; (f) gave their child all required vaccinations; and (g) took their child to a child health examination. If a mother in a CCT project township completed all CCT activities, she would receive about 1000 Chinese yuan (equivalent to 154 US dollars). This is a relatively large sum of money in the study area, given that the average annual income in 2013 was approximately 1500 yuan per capita [7].

### Study design

Our study is not, strictly speaking, a cluster randomized control design but, instead, uses a quasi-experimental design to make a comparison between those who received the treatment and those who did not receive the treatment in two dimensions. One dimension of the comparison is between village, and the other is within village.

For the between-village comparison, we received access to 25 CCT townships, including nine townships from four counties in Gansu and 16 townships from five counties in Sichuan, an average of about three townships per county. One treatment village was randomly selected in each treatment township to generate a total set of 25 treatment villages. The corresponding 50 comparison villages were selected from 50 comparison townships that had similar ethnic, social, economic, and infrastructural characteristics to the treatment towns. A series of control variables were used to select comparison villages, including ethnicity (as measured by the share of Han ethnicity in villages), total village population, nature of the local township road (as measured by the presence of a paved road from the township to the village), share of families who were receiving income support or welfare, and average travel time from the village office to the township health center. Thus, in the study, there were two comparison villages (and sets of respondents) for every treatment village.

For the within-village comparison, we adopted three groups to present the status of the eligibility of CCT programs: a fully eligible group (women who were pregnant during the CCT implementation), a partially eligible group (women with newborns during the CCT implementation), and a non-eligible group (women who had delivered their babies before the CCT implementation). We thus can compare the differences among these three groups within a village.

**Fig. 1:**
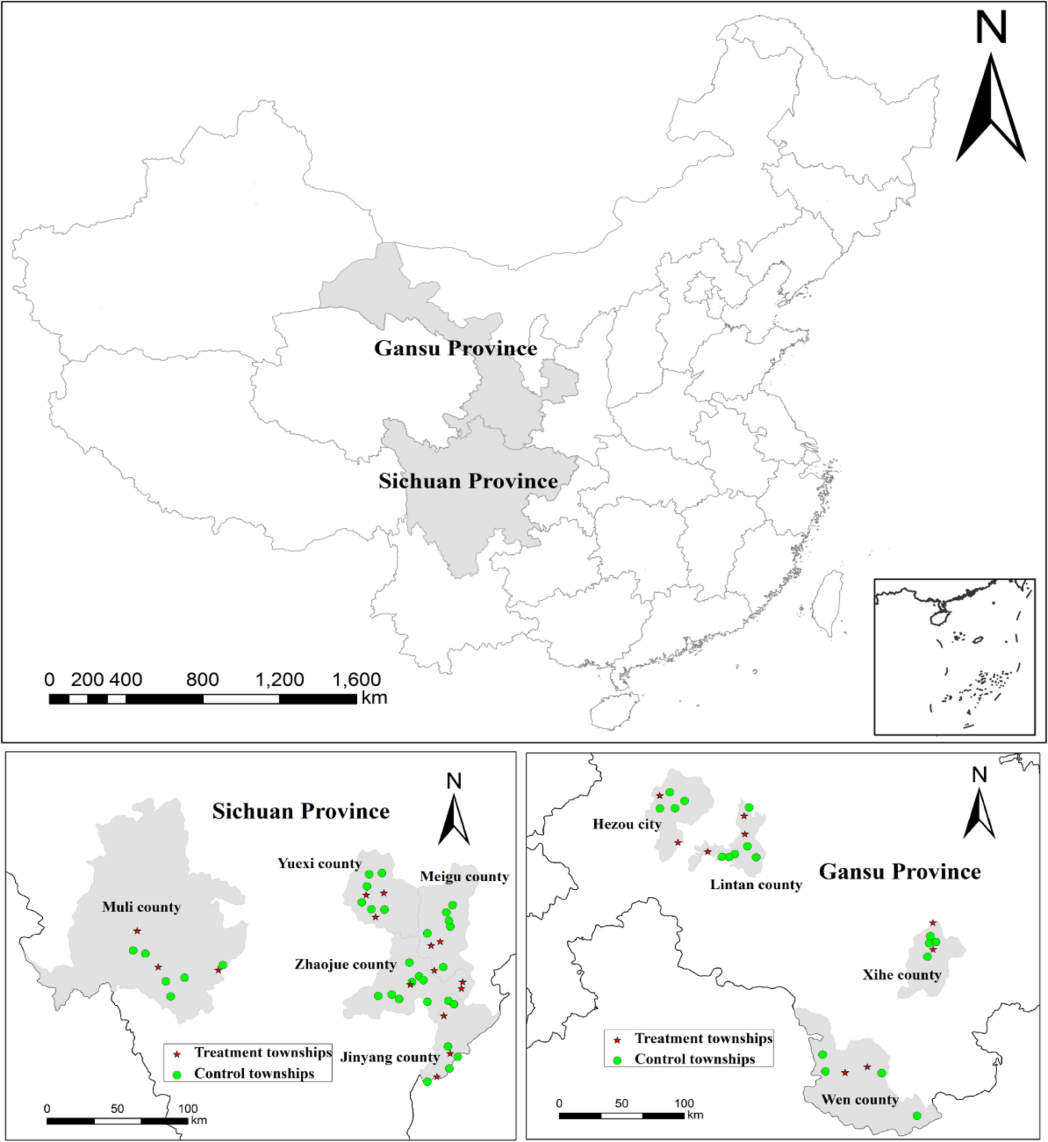
Maps of evaluation areas in poor rural areas of Western China, 2014

The sample villages were selected (and overall sampling protocol was implemented) in fall 2014, 18 months after the launch of the CCT program (as indicated above in spring 2013). Figure 1 depicts the location of the sample townships, counties, and provinces.

### Study sample

Our sampling frame worked as follows. First, we went to the nine CCT project counties from the Sichuan and Gansu provinces. From the five counties in Sichuan, we went to 16 townships that were offering the CCT program (treatment townships) and 32 that were not (comparison townships). From the four counties in Gansu, we went to nine CCT towns and 18 non-CCT towns.

To choose the sample villages and households, we followed a pre-specified protocol that consisted of four steps. The first step was to take a between-village matching strategy. To do so, we randomly chose a set of treatment villages from among the 25 treatment townships that would contain households eligible to participate in the CCT program. After the treatment villages were randomly chosen, the second step involved choosing a set of comparison villages from among the 50 comparison townships. To improve the probability of having a good match, we chose two comparison villages for each treatment village. The assumption of our sampling strategy was that the two comparison villages, by nature of their proximity to the treatment villages, were likely to be close matches.

To select the comparison villages, we used secondary township-level and village-level statistics. Utilizing all available variables noted above for each village in each township, we identified one village from within each of the two comparison townships that were similar to our treatment villages. No significant differences were found between the comparison and the treatment villages (Table 1). This means that each of the two comparison villages were statistically similar to the treatment villages in terms of the relevant township-level and village-level characteristics.

**Table 1:** Characteristics of sample households by treatment status (*N* = 1522)

Observable characteristics	All sample	CCT village			Comparison village			***p*** from test of difference		
		FE	PE	IE	FE	PE	IE	H0: (2) = (5)	H0: (3) = (6)	H0: (4) = (7)
	(1)	(2)	(3)	(4)	(5)	(6)	(7)	(8)	(9)	(10)
***Child characteristics***										
(1) Age, (months)	12.3 (5.9)	6.3 (2.7)	12.7 (2.3)	18.5 (4.0)	6.2 2.7)	12.6 (2.3)	18.7 (3.7)	0.52	0.64	0.57
(2) Girl, (%)	726 (47.7)	82 (47.1)	75 (42.9)	63 (40.9)	186 (52.7)	168 (49.6)	152 (46.5)	0.23	0.15	0.25
(3) First pregnancy, (%)	464 (30.5)	54 (31.0)	55 (31.4)	41 (26.6)	108 (30.6)	103 (30.4)	103 (31.5)	0.92	0.81	0.28
***Mother characteristics***										
(4) Age, (years)	27.7 (6.0)	27.6 (6.4)	27.9 (5.9)	28.8 (6.3)	26.9 (5.9)	27.4 (6.0)	28.1 (5.7)	0.21	0.38	0.23
(5) Non-Han ethnicity,(%)	1010 (66.4)	113 (64.9)	111 (63.4)	96 (62.3)	242 (68.6)	234 (69.0)	214 (65.4)	0.41	0.20	0.51
(6) Junior high school and above education, (%)	387 (25.4)	49 (28.2)	45 (25.7)	42 (27.3)	93 (26.3)	82 (24.2)	75 (22.9)	0.66	0.71	0.30
(7) Farmer or housewife, (%)	1352 (88.8)	159 (91.4)	154 (88.0)	129 (83.8)	323 (91.5)	302 (89.1)	284 (86.9)	0.96	0.71	0.37
***Household characteristics***										
(8) Number of children aged 0–5 years old	1.4 (0.6)	1.4 (0.6)	1.4 (0.6)	1.4 (0.6)	1.5 (0.7)	1.4 (0.6)	1.4 (0.6)	0.82	0.74	0.83
(9) Travel time from household to THC^a^ more than 1 h,(%)	180 (11.8)	15 (8.6)	22 (12.6)	15 (9.7)	38 (10.8)	46 (13.6)	44 (13.5)	0.44	0.75	0.25
(10) Subsistence allowance recipients, (%)	416 (27.3)	51 (29.3)	45 (25.7)	48 (31.2)	89 (25.2)	96 (28.3)	87 (26.6)	0.32	0.53	0.30
(11) Households ranked the poorest quartile in terms of fixed assets^b^, (%)	469 (30.8)	46 (26.4)	45 (25.7)	44 (28.6)	117 (33.1)	105 (31.0)	112 (34.3)	0.12	0.22	0.22
(12) Number of observations	1522	174	175	154	353	339	327			

Notes:

The results are from 1522 women’s interviews from the survey in 2014. The number of observations is presented in the case of binary variables or the mean in the case of continuous variables; percentage or standard deviations are presented in parentheses. *FE* fully eligible for the CCT program; *PE* partially eligible; *IE* ineligible

^a^: Township health center that is the nearest health center that offers maternal and child health services

^b^: Fixed assets are calculated by the method of principal component analysis. After extraction of the principal component, the quartile is used to divide the fixed asset that a household possesses into four levels, including the first quartile (the poorest), the second quartile (poor), the third quartile (rich), and the fourth quartile (the richest)

*Source: authors’ survey*

The sample included 25 treatment villages and 50 comparison villages, for a total of 75 study villages. The sample villages were selected, and the overall sampling protocol was implemented in fall 2014. The timing of the sample selection was carried out 18 months after the launch of the CCT program, which was spring 2013.

For the third step of the sample selection protocol, we chose study households in the treatment and comparison villages. The goal was to choose three different types of households. The first type was termed Fully Eligible (FE) households, meaning that the mother became pregnant after the implementation of the CCT program in the treatment villages. This means that she would have been able to take advantage of all services offered by the program. The second type was termed Partially Eligible (PE) households, wherein the mother became pregnant prior to the launch of the CCT program but did not deliver her child until after its launch in the treatment villages. This means she would have been able to access some, but not all, program services. The third type was termed Ineligible (IE) households, meaning that the mother became pregnant and delivered her child prior to the launch of the CCT program. This would have barred her from being able to access its services even though she was in a CCT treatment village.

To select these households, we went to each village and consulted the roster of all babies from the village doctor or the women’s representative. We grouped babies into three types based on their dates of birth. Within each type of household, we randomly selected seven babies and their mothers to become our sample households. As such, we selected 21 households per village. At the time of the final evaluation survey, FE households had babies who were between 3 and 12 months old; PE households had babies between 13 and 18 months old; and IE households had babies aged 19 to 24 months. Thus, at the time of the launch of the CCT program, IE households had babies who were between 1 and 6 months of age, whereas PE and FE households had mothers who were either pregnant or not pregnant yet, respectively. For clarity of exposition, we further classify households from treatment villages as FE treatment households, PE treatment households, and IE treatment households. Likewise, households from comparison villages are classified as FE comparison (or control) households, PE comparison households, and IE comparison households, matched by the same age period in the fourth step of our analysis.

**Fig. 2:**
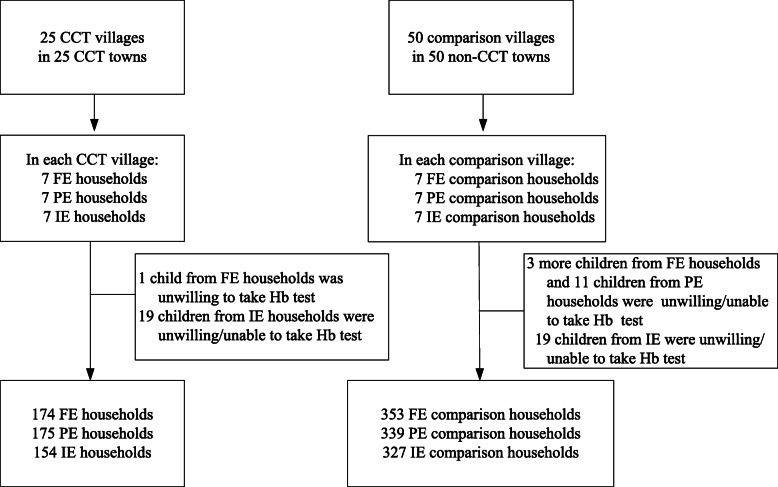
CCT program assessment profile

In summary, the sample included 21 households (7 FE, 7 PE, and 7 IE treatment households) in each of the 25 treatment villages and 21 households (7 FE, 7 PE, and 7 IE comparison households) in each of the 50 comparison villages. In total, the sample included 25 treatment villages and 50 comparison villages, for a total of 75 study villages. In addition, 21 households in each target village were selected based on their eligibility status for the CCT program. The assessment profile of this CCT program is depicted in Fig. 2.

### Data collection

The research team conducted the survey in October 2014 in Sichuan Province and November 2014 in Gansu Province. The survey comprised five modules that were designed to meet our objectives of measuring the impact of the CCT program on the outcomes of interest: uptake of MCH services, knowledge of the mother about MCH issues, health outcomes of the child, participation in and receipt of the CCT payments in the CCT eligible households in the treatment villages, and information on individual and family characteristics that we used as control variables in the analysis.

The first module involved the collection of information needed to assess the uptake of MCH services. This included asking whether the caregiver of the child had participated in antenatal check-ups, in-hospital delivery, postpartum checkups, child physical checkups, or child vaccinations. Using our data, we constructed seven measures of MCH service uptake: any antenatal care visit made (1 = yes, 0 = no); baby delivered in hospital (1 = yes, 0 = no); any postpartum care visit made (1 = yes, 0 = no); early breastfeeding (defined as a practice of a woman who begins to breastfeed within one hour after delivery) (1 = yes, 0 = no); exclusive breastfeeding (defined as a characteristic of a woman who fed her child for 6 months with only breast milk, without supplementing any complementary foods or infant formula) (1 = yes, 0 = no); compliance rate of child physical examinations (%); and compliance rate of child vaccinations (%). The compliance rates of child physical examinations and vaccinations were calculated based on the requirements of the national standards of basic public health services, which has a required schedule of physical examinations and vaccinations that a child should receive from birth to 6 years old. Therefore, the compliance rate is the actual frequency of physical examinations and vaccinations divided by the frequency of the national standards.

The second module concerned the assessment of the mother’s knowledge of the MCH services that she was asked to take her child to as well as knowledge of infant nutrition. In this knowledge scale, there was a total of 22 items, scored by giving the respondent one point for each correct answer; thus, a mother with complete knowledge would score 22 points. Appendix A1 provides an English translation of the knowledge test [see Appendix Table A1 in the Additional file 1]. Using our data, we constructed five measures of the mother’s knowledge about MCH services: total score on the 22-item knowledge test (full = 22 points), at least 60% of the 22-item knowledge test correct (1 = yes, 0 = no), score on items related to maternal care (full = 8 points), score on items related to child nutrition (full = 6 points), and thinking that child physical exams were necessary (1 = yes, 0 = no).

In the third module, each sample child in the treatment and comparison groups received physical examinations. Trained nurses, as part of the survey team, collected data on three indicators of health outcomes for each child. The three measures included hemoglobin concentrations, height, and weight. Hemoglobin levels were measured using HemoCue Hb 201+ systems (HemoCue Inc., Angelholm, Sweden). Following international standards for our sample age group, we defined anemia as a hemoglobin count of less than 110 g per liter [27]. Height and weight measurements were obtained following the World Health Organization (WHO) standard protocol. The children were measured in light clothing without shoes, hats, or accessories. Height was measured using a standard tape measure. Weight was measured with a calibrated electronic scale (Tanita HD-388, Japan). The nursing team was trained to set up the weighing station on level ground to ensure accuracy of the equipment. The anthropometric data were used to develop standard indicators of child development, such as length-for-age *Z*-scores (LAZ) and weight-for-length *Z*-scores (WLZ), based on international standards [28]. Using our data, we constructed four measures of child health outcomes, following WHO guidelines: low birth weight (1 = less than 2500 g, 0 = 2500 g or more), anemia (1 = hemoglobin less than 11.0 g/dl, 0 = 11.0 g/dl or more), stunted growth (1 = LAZ less than − 2 standard deviations, 0 = − 2 or more), and wasting (1 = WLZ less than − 2 standard deviations, 0 = − 2 or more).

Although all other modules were administered to all sample households, regardless of their eligibility status, the fourth was not. For households in the treatment groups, namely, FE and PE treatment households, we had one extra module in which enumerators asked detailed information about their participation in the CCT program. Specifically, enumerators asked whether the mother registered for the program. Mothers were also asked to report the amount of cash that they had received for participating in the CCT MCH activities.

The fifth module of the survey was designed for the collection of information on various factors, statistically, *controls* that might directly or indirectly affect the uptake of MCH services or health outcomes. The survey contained items for mothers about their child’s age, gender, ethnicity, gestational age, and pregnancy order. Enumerators also quizzed mothers about their own characteristics, including age, education, ethnicity, and occupation. A final set of items concerned overall household characteristics, including distance from home to the township health center in terms of kilometers and travel time, and the nature of each household’s durable assets.

### Statistical analysis

A power analysis was conducted based on one of the main outcomes: the rates of hospital delivery. With the power of 0.8 to detect a difference in hospital delivery rates between the treatment and control groups in a cluster controlled trial, a suitable sample size depends on the number of children per village, number of villages, and probability of hospital delivery in treatment villages and controlled villages at a 95% plausible interval. According to our study design, with one CCT village and two control villages, the total number of villages was 75. Based on previous studies [29], we assumed that hospital delivery rates were 50% in control villages and 60% in treatment villages. We then assumed a 95% plausible interval of 0.4 to 0.75. On the basis of these parameters, we calculated that we required 18 women per village. Considering the possible sample loss and assumed impacts, we added three women to each village to overpower the study when the budget allowed.

All statistical analyses were performed using STATA 12.0 (StataCorp, College Station, Texas, USA); *p*-values below 0.05 were considered statistically significant. We reported coefficients and 95% confidence intervals (CIs) for all main variables of interest. Comparisons between the treatment and comparison groups for all outcomes by subgroup populations were assessed using *t*-tests or chi-square tests.

To examine the effect of the CCT program on the uptake and knowledge of MCH services as well as on child health status, the evaluation used two dimensions of variation, i.e., between-village analysis (Evaluation Strategy 1) and within-village analysis (Evaluation Strategy 2). The first is cross-sectional and comes from a comparison of households with the same eligibility but from villages with different treatment status, namely, CCT treatment villages versus non-CCT comparison villages, utilizing Evaluation Strategy 1. Under Strategy 1, we estimated the impact of the CCT program, using the following least squares regressions model:

1 $$ {\mathrm{Y}}_{\mathrm{i}}=\upalpha +{\upbeta \mathrm{CCT}}_{\mathrm{i}}+{\upgamma \mathrm{X}}_{\mathrm{i}}+{\upvarepsilon}_{\mathrm{i}} $$

*Y*_*i*_ is the outcome of interest for household *i*, including uptake of MCH services, knowledge of MCH services, and health status of children; *CCT*_*i*_ is a dummy variable that indicates whether a household comes from a CCT village, which makes *β* the parameter of interest; and *X*_*i*_ is a vector of covariates that are included to capture the characteristics of children, mothers, and households. In all cases, we adjusted standard errors for clustering at the township level, using a cluster-corrected estimator.

The second dimension of variation is temporal and comes from comparing households that are fully or partially eligible (FE/PE households) against those households that are ineligible (IE households) for this CCT program under Evaluation Strategy 2. In this evaluation strategy, we estimated the impact of the CCT program, using the following least squares regression model:

2 $$ {\mathrm{Y}}_{\mathrm{i}}=\upalpha +{\upbeta \mathrm{Eligibility}}_{\mathrm{i}}+{\upgamma \mathrm{X}}_{\mathrm{i}}+{\upvarepsilon}_{\mathrm{i}} $$

Note that the only difference between eqs. (1) and (2) is that we replaced *CCT*_*i*_ with the dummy variable *Eligibility*_*i*_, indicating whether a household is fully or partially eligible (FE/PE households). The rest of the variables are the same as described in eq. (1). Together, Strategies 1 and 2 consist of a comparison of households whose children were born at different times (before, during, or after the launch of the CCT program) and by CCT status. The CCT program can be considered the treatment, and our sample households were divided into two treatment groups and a comparison/control group. The treatment groups include (a) the FE households in the CCT villages and (b) the PE households in the CCT villages. The comparison group includes all of the households in the non-CCT comparison villages (FE, PE, and IE households) as well as the IE households in the CCT villages.

A within-village difference for the first-stage difference and between-village but within-township difference for the second stage allows us to apply the difference-in-difference strategy to evaluate the effect of CCT on utilization of maternal health services and health outcomes. The baseline values for the difference-in-difference analysis were generated with the data of households that were ineligible for the CCT program.

We supplemented our intention-to-treat (ITT) multivariable analysis described above by examining the average-treatment-effects-on-the-treated (ATT analysis) to measure the impact on outcomes among the subpopulation of households that had heard about the CCT program. This allowed us to control for any confounding due to non-compliance, which we define as use of MCH services without receiving a monetary transfer. For the ATT analysis, we utilized an instrumental variable (IV) approach [30], in which the treatment assignment (receiving CCT information or not) was used to account for observed compliance or receiving a monetary transfer for using MCH services. This analysis is based on the assumption that the only reason for a woman in a CCT village to not receive a monetary transfer for using an MCH service is that she was unaware of the CCT program. The IV approach allows us to measure the average effect of treatment on the use of MCH services, mother’s knowledge, and child health outcomes among the subpopulation of households that knew about the program and, thus, control for confounding due to non-compliance. The ATT analyses for the continuous outcome measures were performed using STATA’s ivreg model. The ATT analyses for the binary outcome measures were performed using STATA’s ivprobit model. In estimating both models, we clustered the standard errors at the village level. STROBE [31], and BMC guidelines were used to organize our paper.

## Results

### Participants’ characteristics

A total of 1522 households (mother-baby pairs) were enrolled in our study. In total, there were 503 households in the 25 treatment villages. Of these, 349 were treatment households (174 FE households, 175 PE households), and 154 were comparison households (IE households). Likewise, there were 1019 households in the 50 comparison villages (non-CCT villages). Of these, 353 were FE households, 339 were PE households, and 327 were IE households.

Table 1 presents the results for the observable characteristics of the children, mothers, and households across different treatment and comparison groups. As seen in the table, the mean age of all of the children in the study was 12.3 months (SD = 5.9), and 47.7% of the children were girls. The mean age of the mothers was 27.7 years. Nearly 70% of the mothers were of non-Han ethnicity, and only 25% of the mothers received a junior high school or above level of schooling. Out of the total number of households, 11.8% needed more than one hour to travel to the township health centers.

For the integrity of the evaluation, the observable characteristics across treatment and comparison households are balanced (Columns 2 to 10). In the case of all of the individual variables, the *p*-values were above 0.05. Below, we present the comparisons between CCT and non-CCT villages, including uptake of MCH services, impacts on mother’s knowledge, and impacts on health outcomes.

### Uptake of MCH services

#### FE households

A comparison of FE households in the CCT and non-CCT villages showed that two out of the seven uptake measures were significant (Table 2; Rows 1 and 6; Columns 1, 4, and 7; both *p*-values were less than 0.05). Specifically, more women in the FE households in the CCT villages made at least one prenatal care visit (85%) than did those in the comparison villages (78%). Although the differences were significant (*p* = 0.04), the magnitude of the difference was modest (7%). Mothers in FE households in CCT villages also took their children for post-natal physical examinations at a higher rate (33%) than did those in the comparison villages (23%). As in the case of prenatal visits, although the difference was significant (*p* = 0.01), the size of the difference was relatively small (10%). In the case of the other program healthcare activities—delivery in hospital, postpartum care visits, early breastfeeding, exclusive breastfeeding, and compliance with child vaccinations—there were no statistically significant differences in the rates of participation between the treatment and comparison villages.

**Table 2:** Utilization of MCH services, knowledge, and health outcomes by treatment status (*N* = 1522)

Dependent variable	CCT village			Comparison village			***p*** value	
	FE	PE	IE	FE	PE	IE	H0:(1) = (4)	H0:(2) = (5)
	(1)	(2)	(3)	(4)	(5)	(6)	(7)	(8)
***Utilization of MCH services***								
(1) Made any antenatal care visit, (%)	148 (85.1)	143 (81.7)	126 (81.8)	274 (77.6)	254 (74.9)	254 (77.7)	0.04	0.08
(2) Delivery in hospital, (%)	119 (68.4)	123 (70.3)	103 (66.9)	235 (66.6)	209 (61.7)	180 (55.0)	0.68	0.05
(3) Made any postpartum care visit, (%)	84 (48.3)	99 (56.6)	82 (53.2)	250 (42.5)	132 (39.2)	135 (41.3)	0.21	0.00
(4) Early breast feeding, (%)	56 (32.2)	65 (37.1)	51 (33.1)	113 (32.0)	106 (31.9)	106 (32.4)	0.97	0.23
(5) Exclusive breast feeding, (%)	52 (51.0)	82 (47.1)	88 (58.7)	76 (39.2)	150 (44.5)	154 (48.0)	0.05	0.57
(6) Compliance rate of physical examination, (%)^c^	58 (33.4)	51 (29.0)	35 (22.9)	81 (22.9)	52 (15.2)	49 (14.9)	0.01	0.00
(7) Compliance rate of child vaccinations, (%)^c^	136 (78.1)	128 (73.2)	122 (79.5)	258 (73.0)	237 (70.0)	239 (73.2)	0.05	0.30
***Mother’s Knowledge***								
(8) Total knowledge score (full = 22)	10.9 (4.4) ^b^	11.0 (4.1)	11.1 (4.0)	9.9 (4.1)	10.1 (4.2)	9.9 (4.3)	0.01	0.02
(9) Got at least 60% correct, (%)	56 (32.2)	57 (32.6)	47 (30.5)	76 (21.5)	90 (26.5)	82 (25.1)	0.01	0.15
(10) Score on maternal care (full = 8)	4.8 (1.9)	5.0 (1.7)	4.8 (1.8)	4.6 (1.9)	4.5 (2.1)	4.3 (2.1)	0.31	0.01
(11) Score on child nutrition (full = 6)	2.8 (1.4)	2.7 (1.4)	2.4 (1.3)	2.3 (1.3)	2.5 (1.3)	2.6 (1.4)	0.00	0.04
(12) Thinking child physical examination necessary, (%)	28 (16.1)	34 (19.4)	14 (9.1)	26 (7.5)	32 (9.5)	27 (8.3)	0.02	0.00
***Health outcome***								
(13) Low birth weight, (%)	6 (3.5)	14 (8.1)	7 (4.8)	20 (5.7)	24 (7.0)	20 (6.0)	0.31	0.67
(14) Anemia, (%)	51 (38.9)	76 (55.5)	48 (37.5)	116 (42.3)	129 (49.0)	87 (34.8)	0.52	0.22
(15) Stunted growth, (%)^a^	16 (9.4)	17 (9.8)	27 (17.8)	26 (7.5)	47 (14.0)	68 (20.8)	0.47	0.18
(16) Wasting, (%)^b^	5 (2.9)	7 (4.0)	1 (0.6)	8 (2.3)	10 (2.9)	10 (3.1)	0.67	0.53
(17) Number of observations	174	175	154	353	339	327		

Notes:

The number of observations is presented in the case of binary variables or the mean in the case of continuous variables; percentage or standard deviations are presented in parentheses. *FE* fully eligible for the CCT program; *PE* partially eligible; *IE* ineligible. Compliance rates of physical examinations and child vaccinations are calculated by the requirements of the national standards of basic public health services by child age

^a^: Stunted growth: length-for-age Z-scores are less than −2 standard deviations

^b^: Wasting: weight-for-height Z-scores are less than −2 standard deviations

*Source: authors’ survey*

When the ITT model was applied to Evaluation Strategy 1 for FE mothers (Table 3; Rows 1–7; Columns 1–3), all of the point estimates were positive, and all of the 95% confidence intervals overlapped zero. After holding all of the control variables constant except for compliance (which we do below in the ATT model), the CCT intervention did not have a large impact on the uptake of MCH services.

**Table 3:** Intention-to-treat (ITT) analysis for the effects of CCT treatment (*N* = 1522)

	FE-CCT Versus FE-comparison^b^			PE-CCT Versus PE-comparison^b^		
Dependent variable	*β*	95% CI	***p***	*β*	95% CI	***p***
	(1)	(2)	(3)	(4)	(5)	(6)
***Uptake of MCH services***						
(1) Any antenatal examination (%)	0.44	−0.30, 1.18	0.24	0.41	− 0.27, 1.09	0.24
(2) Hospital delivery (%)	0.01	−0.72, 0.74	0.98	0.38	− 0.36, 1.11	0.38
(3) Postpartum visits (%)	0.23	−0.30, 0.76	0.40	0.84	0.27, 1.41	0.00*
(4) Early breastfeeding (%)	0.02	−0.08, 1.19	0.09	0.06	−0.43, 0.55	0.81
(5) Exclusive breastfeeding (%)	0.55	−0.00, 0.95	0.05	0.05	−0.44, 0.54	0.84
(6) Compliance rate of physical examination, (%)^a^	0.10	−0.01, 0.21	0.06	0.13	0.02, 0.24	0.02*
(7) Compliance rate of child vaccinations, (%)^a^	0.04	−0.03, 1.15	0.25	0.02	−0.06, 0.10	0.57
***Mother’s knowledge***						
(8) Total knowledge scores (full = 22)	0.91	−0.19, 2.00	0.10	0.82	−0.07, 1.72	0.07
(9) Got at least 60% correct (%)	0.58	0.02, 1.14	0.04*	0.30	−0.26, 0.85	0.29
(10) Score on maternal care (full = 8)	0.15	−0.34, 0.64	0.54	0.48	0.08, 0.89	0.02*
(11) Score on child nutrition (full = 6)	0.39	0.08, 0.70	0.01*	0.22	−0.04, 0.49	0.10
(12) Thinking child physical examination necessary (%)	0.66	−0.07, 1.40	0.08	0.87	0.20, 1.54	0.01*
***Child health outcomes***						
(13) Low birth weight (%)	−0.4	−1.31, 0.52	0.40	0.23	−0.53, 0.99	0.55
(14) Anemia (%)	−0.19	−0.68, 0.30	0.45	0.27	−0.22, 0.77	0.28
(15) Stunted growth (%)^b^	0.19	−0.49, 0.88	0.58	−0.42	−1.20, 0.36	0.29
(16) Wasting (%)^c^	0.13	−1.23, 1.48	0.85	0.36	−0.89, 1.62	0.57

Notes:

Linear and logistic regression are used to analyze CCT’s impact on uptake of health services, mother’s knowledge, and child health outcomes. Covariates include child’s age, gender, low birth weight, premature birth, birth order, mother’s ethnicity, education, occupation, number of children, whether the family received social security support, distance from household to township heath center, travel time from household to township health center, and household fixed assets. Standard errors are clustered at the town level

Appendix Table A4 [see Additional file 1] shows details on regression specification. *FE* fully eligible for the CCT program; *PE* partially eligible; *IE* ineligible

^a^: Compliance rates of physical examinations and child vaccinations are calculated by the requirements of the national standards of basic public health services by child age

^b^: Stunted growth: length-for-age Z-scores are less than − 2 standard deviations

^c^: Wasting: weight-for-height Z-scores are less than − 2 standard deviations. *p < 0.05

Source: authors’ survey

#### PE households

When we compared PE households in CCT and non-CCT villages, using both the descriptive and ITT analyses, the overall results are similar to those for FE mothers. The data suggested that the impact of the CCT program was modest for women who were only partially eligible (PE mothers). The descriptive data showed that two out of the seven uptake measures were significant (Table 2; Rows 3 and 6; Columns 2, 5, and 8; both *p*-values were less than 0.05). The findings indicated that 56.6% of mothers in the CCT villages attended postpartum care visits, but only 39.2% of those in the non-CCT villages attended (*p* < 0.001). Similar to FE household comparisons, there were also statistical differences in the compliance rate of child physical examinations between PE households (29.0% in the CCT villages compared to 15.2% in the non-CCT villages, *p* < 0.001; Table 2; Row 6; Columns 2, 5, and 8). In the case of all other indicators, there were no differences between PE treatment village mothers and PE comparison village mothers.

The results from the ITT analysis, using PE mothers, were consistent with those of the descriptive data. When compared to PE mothers in non-CCT villages, PE mothers in CCT villages were more likely to attend any postpartum care visit (*β* = 0.84, *CI* = 0.27 to 1.41; *p* < 0.001) and to take their children to more health checkups (*β* = 0.13, *CI* = 0.02 to 0.24; *p* = 0.02) (Table 3; Rows 3 and 6; Columns 4–6). The ITT results did not show any impact of the CCT program on any of the other five MCH services. Indeed, when comparing either the FE mothers or the PE mothers in the treatment and comparison villages, the impact of the CCT program on MCH service uptake was quite small.

### Impacts on mother’s knowledge

The knowledge of MCH issues is low among all women in the sample areas, in general, regardless of being in a treatment or comparison group (Table 2; Rows 8–12). The women from CCT villages, however, had significantly higher total knowledge scores than did those from comparison villages (Table 2; Row 8 Columns 1–8). The same results can be found in regard to knowledge of child nutrition (Table 2; Row 11, Columns 1–8). Less than 20% of the women believed that child health examinations were necessary for their children’s health, and a statistically significant difference was found between CCT and comparison villages (Table 2; Row 12, Columns 1–8).

When comparing either FE or PE women in CCT treatment villages with their counterparts in the comparison villages, using descriptive statistics, there were statistically significant differences between a number of measures (Table 2; Rows 8–12; Columns 7 and 8). Using either the descriptive statistics or the ITT approach, however, the team found that, when they estimated the impact of the CCT program on mothers’ knowledge about MCH services, there was only a small effect (Tables 2 and 3; Rows 8–12). The impact of the CCT program on the knowledge of mothers is, therefore, similar to the impact of the CCT program on MCH service uptake, as there was a statistically significant, but small, impact.

Two observations moderate any attempts to claim that the CCT program did any more than modestly improve the knowledge of the women participants. First, in the descriptive analysis, the magnitudes of the differences between the FE and PE respondents in CCT treatment and non-CCT comparison villages were small. At most, the CCT treatment village women improved their knowledge by 1 point (out of 22 for the overall knowledge scale). The measured differences in magnitudes for all of the other analyses were even smaller. Second, for the ITT analysis, which held equal the characteristics of the child, mother, and family, most of the measured impacts of the CCT intervention on either FE or PE women became insignificant.

### Impacts on health outcomes

There was almost no effect on incidences of low birth weight, anemia, stunted growth, or wasting. The descriptive analysis, using Evaluation Strategy 1, found no improvement on any of the outcome measures between either the FE children or PE children in the treatment and comparison villages (Table 2; Rows 13–16; Columns 7 and 8). Likewise, the ITT analysis did not detect any improvements for any of the outcomes when considering the effect of the CCT intervention on FE or PE children in the treatment villages compared to those in the comparison villages.

### Robustness of results to alternative evaluation strategy

Using Evaluation Strategy 2, we found that there was only a very modest effect on MCH service utilization; the study found no difference in maternal knowledge. There also was almost no positive effect of the CCT program on health outcomes of children according to the descriptive and ITT analyses (Tables 4 and 5).

**Table 4:** Utilization of MCH services, knowledge, and health outcomes by eligibility status in CCT village

Dependent variable	CCT village			***p*** value	
	FE	PE	IE	H0:(1) = (3)	H0:(2) = (3)
	(1)	(2)	(3)	(4)	(5)
***Utilization of MCH services***					
(1) Made any antenatal care visit, (%)	148 (85.1)	143 (81.7)	126 (81.8)	0.43	0.98
(2) Delivery in hospital, (%)	119 (68.4)	123 (70.3)	103 (66.9)	0.77	0.51
(3) Made any postpartum care visit, (%)	84 (48.3)	99 (56.6)	82 (53.2)	0.37	0.55
(4) Early breast feeding, (%)	56 (32.2)	65 (37.1)	51 (33.1)	0.86	0.45
(5) Exclusive breast feeding, (%)	52 (51.0)	82 (47.1)	88 (58.7)	0.23	0.05
(6) Compliance rate of physical examination, (%)^c^	58 (33.4)	51 (29.0)	35 (22.9)	0.01	0.21
(7) Compliance rate of child vaccinations, (%)^c^	136 (78.1)	128 (73.2)	122 (79.5)	0.86	0.22
***Mother’s Knowledge***					
(8) Total knowledge score (full = 22)	10.9 (4.4) ^b^	11.0 (4.1)	11.1 (4.0)	060	0.79
(9) Got at least 60% correct, (%)	56 (32.2)	57 (32.6)	47 (30.5)	0.75	0.69
(10) Score on maternal care (full = 8)	4.8 (1.9)	5.0 (1.7)	4.8 (1.8)	0.79	0.35
(11) Score on child nutrition (full = 6)	2.8 (1.4)	2.7 (1.4)	2.4 (1.3)	0.88	0.83
(12) Thinking child physical examination necessary, (%)	28 (16.1)	34 (19.4)	14 (9.1)	0.09	0.01
***Health outcome***					
(13) Low birth weight, (%)	6 (3.5)	14 (8.1)	7 (4.8)	0.59	0.27
(14) Anemia, (%)	51 (38.9)	76 (55.5)	48 (37.5)	0.81	0.00
(15) Stunted growth, (%)^a^	16 (9.4)	17 (9.8)	27 (17.8)	0.03	0.04
(16) Wasting, (%)^b^	5 (2.9)	7 (4.0)	1 (0.6)	0.13	0.05
(17) Number of observations	174	175	154		

Notes:

The number of observations are presented in the case of binary variables or mean in the case of continuous variables; percentage or standard deviations are presented in parentheses. *FE* fully eligible for the CCT program; *PE* partially eligible; *IE* ineligible. The compliance rates for physical examinations and child vaccinations are calculated by the requirements of the national standards of basic public health services by child’s age

^a^: Stunted growth: length-for-age *Z*-scores are less than −2 standard deviations

^b^: Wasting: weight-for-height *Z*-scores are less than −2 standard deviations

*Source: authors’ survey*

**Table 5:** Intention-to-treat (ITT) analysis for the effects of CCT treatment in CCT villages

Dependent variable	FE-CCT Versus IE-CCT^b^			PE-CCT Versus IE-CCT		
	*β*	95% CI	***p***	*β*	95% CI	***p***
	(1)	(2)	(3)	(4)	(5)	(6)
***Uptake of MCH services***						
(1) Any antenatal examination (%)	0.14	−0.26, 0.55	0.48	−0.1	− 0.75, 0.55	0.77
(2) Hospital delivery (%)	0.10	−0.44, 0.63	0.72	0.26	−0.34, 0.87	0.40
(3) Postpartum visits (%)	0.28	−1.15, 1.71	0.70	0.27	−0.45, 1.00	0.46
(4) Early breastfeeding (%)	−1.07	−2.77, 0.63	0.23	−0.51	−1.56, 0.54	0.34
(5) Exclusive breastfeeding (%)	−0.06	−1.44, 1.32	0.93	−0.29	−1.04, 0.45	0.44
(6) Compliance rate of physical examination, (%)^a^	0.10	0.02, 0.19	0.02*	−0.05	− 0.03, 0.14	0.22
(7) Compliance rate of child vaccinations, (%)^a^	0.01	−0.08, 0.11	0.76	−0.05	− 0.12, 0.03	0.20
***Mother’s knowledge***						
(8) Total knowledge scores (full = 22)	0.33	−1.51, 2.17	0.71	−0.29	−1.66, 1.09	0.67
(9) Got at least 60% correct (%)	0.66	−0.37, 1.70	0.21	0.06	−0.59, 0.72	0.85
(10) Score on maternal care (full = 8)	0.51	− 0.49, 1.52	0.30	0.28	−0.31, 0.88	0.33
(11) Score on child nutrition (full = 6)	−0.14	− 0.76, 0.49	0.65	− 0.17	− 0.53, 0.19	0.33
(12) Thinking child physical examination necessary (%)	1.97	−0.12, 4.06	0.06	1.40	0.02, 2.79	0.04*
***Child health outcomes***						
(13) Low birth weight (%)	1.03	−1.65, 3.71	0.45	0.68	−0.68, 2.04	0.33
(14) Anemia (%)	0.00	−1.13, 1.13	1.00	0.91	0.25, 1.56	0.01*
(15) Stunted growth (%)^b^	0.23	−1.16, 1.62	0.74	−0.58	−1.67, 0.52	0.30
(16) Wasting (%)^c^	−0.73	−1.46, 0.00	0.05	1.15	0.12, 2.17	0.03*

Notes: Linear and logistic regressions are used to analyze CCT’s impact on uptake of health services, mother’s knowledge, and child health outcomes. Covariates include child’s age, gender, low birth weight, premature birth, and birth order; mother’s ethnicity, education, and occupation; and number of children, whether the family received social security support, distance from household to township heath center, travel time from household to township health center, and household fixed assets. Standard errors are clustered at the town level

^a^: Compliance rates of physical examinations and child vaccinations are calculated by the requirements of the national standards of basic public health services by child’s age

^b^: Stunted growth: length-for-age *Z*-scores are less than − 2 standard deviations

^c^: Wasting: weight-for-height *Z*-scores are less than − 2 standard deviations. *p < 0.05

*Source: authors’ survey*

### Treatment compliance and the results of the ATT analysis

In CCT villages, out of 174 sample FE households that were included in the descriptive and ITT analyses (reported above), only 55.8% reported that they had heard about the CCT program. Out of the 175 sample PE households in CCT villages, only 62.3% reported that they had heard about the program. In total, 59.0% of the women (both FE and PE women) in CCT villages said that they had been informed about the CCT program. Of the women who knew about the CCT program, the proportion who used MCH services and received a monetary transfer is even lower. The overall compliance rate of the CCT program was only 49.9%. Specifically, only 51.1% of the women from FE households received at least one type of MCH services and received a cash transfer. In the PE households, only 48.6% of the women complied.

The results of the ATT analysis (using either evaluation strategy) mirrored the results of the ITT analyses. The CCT intervention had only a small impact on the uptake of MCH services and on improving mother’s knowledge. There was even a smaller (indeed, most likely zero) impact on improving child health outcomes (Table 6).

**Table 6:** Average-treatment-effect-on-the-treated (ATT) analysis for the effects of CCT treatment (*N* = 1522)

Dependent variable	FE-CCT Versus FE-comparison^b^			PE-CCT Versus PE-comparison^b^			FE-CCT Versus IE-CCT^b^			PE-CCT Versus IE-CCT		
	*β*	95% CI	***p***	*β*	95% CI	***p***	*β*	95% CI	***p***	*β*	95% CI	***p***
	(1)	(2)	(3)	(4)	(5)	(6)	(7)	(8)	(9)	(10)	(11)	(12)
***Uptake of MCH services***												
(1) Any antenatal examination (%)	0.12	−0.04, 0.28	0.14	0.10	−0.05, 0.26	0.20	0.03	−0.98, 1.05	0.95	−0.67	−2.42, 1.08	0.44
(2) Hospital delivery (%)	0.01	−0.20, 0.21	0.95	0.11	−0.07, 0.30	0.23	0.93	−0.98, 2.84	0.32	0.44	−0.96, 1.84	0.52
(3) Postpartum visits (%)	0.07	−0.11, 0.25	0.43	0.24	0.08, 0.40	0.00*	0.41	−1.26, 2.08	0.62	0.54	−1.14, 2.22	0.51
(4) Early breastfeeding (%)	0.00	−0.18, 0.18	0.98	0.08	−0.08, 0.25	0.31	−0.26	−1.40, 0.89	0.65	0.46	−1.16, 2.09	0.56
(5) Exclusive breastfeeding (%)	0.18	−0.04, 0.41	0.10	0.02	−0.17, 0.21	0.85	−0.92	−2.78, 0.93	0.32	−0.71	−2.49, 1.08	0.42
(6) Compliance rate of physical examination, (%)^a^	0.18	0.00, 0.37	0.05	0.21	0.04, 0.38	0.01*	0.02	−1.31, 1.36	0.97	−0.21	− 1.73, 1.31	0.78
(7) Compliance rate of child vaccinations, (%)^a^	0.08	−0.05, 0.21	0.21	0.05	−0.07, 0.16	0.41	0.24	−0.60, 1.08	0.55	−0.25	−1.38, 0.88	0.65
***Mother’s knowledge***												
(8) Total knowledge scores (full = 22)	1.65	−0.24, 3.53	0.09	1.32	−0.07, 2.71	0.06	2.08	−8.88, 13.04	0.70	−2.98	−18.71, 12.75	0.70
(9) Got at least 60% correct (%)	0.18	−0.01, 0.36	0.06	0.07	−0.06, 0.21	0.30	0.70	−0.89, 2.30	0.37	0.15	−0.94, 1.23	0.78
(10) Score on maternal care (full = 8)	0.28	−0.59, 1.15	0.53	0.78	0.16, 1.40	0.02*	3.22	−5.90, 12.35	0.47	2.95	−5.25, 11.16	0.46
(11) Score on child nutrition (full = 6)	0.71	0.18, 1.24	0.01*	0.36	−0.05, 0.77	0.09	−0.87	−5.13, 3.40	0.68	−1.81	−6.94, 3.32	0.47
(12) Thinking child physical examination necessary (%)	0.13	−0.02, 0.28	0.09	0.15	0.03, 0.27	0.02*	0.98	−0.92, 2.89	0.30	1.24	−0.68, 3.17	0.20
***Child health outcomes***												
(13) Low birth weight (%)	−0.04	−0.11, 0.04	0.33	0.03	−0.06, 0.12	0.56	0.15	−0.17, 0.48	0.34	0.44	−0.52, 1.41	0.35
(14) Anemia (%)	−0.08	−0.29, 0.13	0.44	0.10	−0.09, 0.29	0.30	−0.02	−2.82, 2.77	0.99	2.00	−2.19, 6.18	0.33
(15) Stunted growth (%)^b^	0.02	−0.07, 0.12	0.59	−0.07	−0.19, 0.05	0.23	0.06	−0.85, 0.98	0.89	−0.66	−2.11, 0.79	0.36
(16) Wasting (%)^c^	0.00	−0.06, 0.07	0.91	0.02	−0.05, 0.08	0.64	−0.03	−0.15, 0.09	0.60	0.16	−0.22, 0.54	0.39

Notes: Instrumental variable analysis is used to measure the impact on outcomes among the subpopulation of households who had heard CCT news. Covariates include child’s age, gender, low birth weight, premature birth, and birth order; mother’s ethnicity, education, and occupation; and number of children, whether the family received social security support, distance from household to township heath center, travel time from household to township health center, and household fixed assets. Standard errors are clustered at the town level

^a^: Compliance rates of physical examinations and child vaccinations are calculated by the requirements of the national standards of basic public health services by child’s age

^b^: Stunted growth: length-for-age *Z*-scores are less than −2 standard deviations

^c^: Wasting: weight-for-height *Z*-scores are less than − 2 standard deviations

*p < 0.05

*Source: authors’ survey*

## Discussion

In this paper, we utilized rigorous impact evaluation approaches on a large sample of women who participated (or did not participate) in a pilot CCT program that sought to incentivize new mothers in two poor regions in Western China to improve their utilization of MCH services, enhance their knowledge, and, ultimately, improve health outcomes. In these areas, the utilization of MCH services and mother’s knowledge are poor overall. Health outcomes of children also are fairly poor. The purpose of the program was to increase the benefit of utilizing MCH services, and this was expected to lead to better knowledge about child health and better child health outcomes.

The data, however, showed that this pilot program had a limited impact. Regardless of the evaluation strategy or the nature of the treatment and control groups, the rate of participation in MCH services did not rise systematically. Mother’s knowledge improved only marginally. Further, health outcomes did not show any sign of improvement. Indeed, throughout the results, from both the descriptive and multivariable analyses, there were instances in which there were significant differences between the treatment of mothers/children in CCT villages and the control mothers/children in non-CCT villages. Nevertheless, in a large proportion of the statistically significant results, the magnitudes of the shifts in outcomes (MCH service utilization, mother’s knowledge, or child health outcomes) were small. That is, the CCT program technically worked, but the effects were so small that they could not be considered meaningful.

In this respect, this pilot project’s relatively minor outcomes differ from those of similar programs implemented in other countries. In other middle- or low-income countries in which CCT programs have been used to help poor women overcome barriers to MCH services, many programs have been shown to improve health. For example, CCT programs have been shown to improve birth weight [16], decrease anemic rates [32], and aid child growth in Mexico [19]. Successful programs in Brazil have reduced overall infant mortality rates [33]. Programs in Nicaragua [34], Ecuador [35], and Colombia [36] have succeeded in increasing child height.

Consistent with the results of this study, however, there also have been CCT programs that did not work. An assessment of a CCT program in Honduras found no effect of such programs on child outcomes [23]. In the Honduran study, it appeared that poor implementation was largely to blame. Another CCT program in Kenya also found no improvement in care. In this case, the poor quality of the healthcare system was at least part of the reason that no improvements in health outcomes were found [24].

In reflecting on the literature, we questioned why the experience with the CCT program in China more resembles that of the CCT programs of the Honduras and Kenya (which did not have an impact) rather than that of successful programs elsewhere—in other words, why was this CCT program so ineffective? Although identifying the precise reason is, unfortunately, beyond the scope of this study, we suspect a number of potential explanations.

One possibility is that the CCT cash transfers might not have been large enough, which would lead to low uptake. Given the way this intervention was designed, however, we doubt this is the case. If a mother had taken advantage of all of the incentivized activities, she would have earned more than 1000 yuan. In these very poor communities, this is a sizeable amount, given that the average annual income in 2013 was approximately 1500 yuan per capita [7]. Thus, this is likely not the only reason for the program’s underperformance.

Another possibility is that travel distances from villages to township health centers may have been prohibitively far. This does not seem likely, however, as only a small share—less than 15%—of target households were more than one hour away from the township health centers. Thus, at least for most mothers, this was unlikely to be a constraint. Likewise, our heterogeneous analysis identified no impacts of travel time from their household to the township health center [see Appendix Table A2 in the Additional file 1]. This result is consistent with the previous research on geographic accessibility in ethnic minority areas of Western China [37].

We also found that mother’s ethnicity and education were associated with the uptake of MCH services and knowledge [see Appendix Table A3 in the Additional file 1]. In China, ethnic minorities face many economic and educational disadvantages stemming from more rural, isolated residences, difficult and mountainous topography, and poor infrastructure connecting these communities to public services. In this study, 66% of the women were members of the Yi or Tibetan ethnic minority groups. These ethnic groups consist mostly of subsistence farmers who live in remote mountainous areas of Sichuan Province, and tend to have low levels of education and little access to formal health care [37]. Previous research has indicated that due to the poor quality but high cost of care, and the cultural differences in birthing practices that cause Yi and Tibetan women discomfort and embarrassment, Yi and Tibetan women may choose to give birth at home [37, 38]. The low uptake of MCH care is also correlated with lower education levels and healthcare knowledge among ethnic minorities [39–42]. Our research similarly suggested that ethnic minority status and education were still important demand-side factors that were barriers to the use of MCH services.

However, these demand-side factors were the exact barriers to MCH service uptake that the CCT program was supposed to overcome. Thus, there must be other factors that influence the effectiveness of the CCT program and explain low levels of improvement that we found. We suspect that problems with the CCT program’s implementation, similar to those encountered in the Honduras study [23], may provide an explanation for the low observed effectiveness of the CCT program. Although the program was supposed to be aggressively promoted, only 60% of the women in the FE or PE households in CCT villages knew about it. Further, although the women were offered a CCT to get MCH services, they might not have believed that they would get paid. In the FE and PE households in the CCT villages, compliance in the CCT program was low: Only about 20% of the women received at least two types of MCH services with monetary transfers, and about 50% of the women received at least one type of MCH service with monetary transfers. Our further ATT analysis showed that uptake of MCH services and knowledge would be significantly improved if women had actually heard about the CCT program. In other words, the use of MCH services and mother’s MCH knowledge would be improved if the CCT program were implemented more effectively.

Another potential reason for this lack of CCT participation may be due to low medical quality and consequent absence of trust in the Chinese medical system. There is a growing body of literature that documents the low quality of health care in rural China [43–45]. Because these studies were conducted in relatively better-off areas of rural China, it is plausible that the quality of the doctors and general health care in these poorer and more remote areas of this study are even worse. Hence, like the study in Kenya [24], it may be that child health outcomes did not improve due to the low quality of health care. This low quality of care undermines mother’s trust, creating a demand-side issue for MCH uptake and weakening the effectiveness of the CCT program.

This study has several limitations. First, due to the implementation schedule of the CCT program, we were unable to collect baseline data on either the treatment or control individuals/villages. The one-year implementation time may not be long enough to track changes in children’s outcomes. As our study focuses primarily on MCH service uptake (which is mostly those services used by the family during the child’s first year of life), however, we believe that the tracking of such outcomes does provide sufficient information to be able to evaluate the impact of this CCT program on the uptake of MCH services. Future research, of course, should continue following children for longer periods of time. Second, in part due to the absence of baseline data, our evaluation design was not a randomized control trial, and CCT villages were not randomly assigned. To do our best to decrease evaluation bias and increase evaluation power, we used a two-pronged evaluation strategy: a between-village matching strategy and a within-village difference-in-difference strategy. These strategies let us compare the changes not only after the CCT program was launched between CCT treatment villages and control villages but also before and after the CCT program was launched within villages.

Another fundamental limitation of this paper is that we are not able to provide a full, empirically based prescription on how policymakers should focus their efforts to try to improve access to MCH services and improve health outcomes. In this paper, we can show only that uptake and knowledge of MCH services are poor and that the pilot CCT program really did not solve the problem. We did spend a lot of time in addressing the potential reasons for this, including poor implementation of the program, an absence of trust in the system, and poor quality health care in general (which may reduce the impact on health outcomes, given access, and undermine interest in the program). The current study, however, does not provide an empirical basis for conclusive findings on what to do in the future. Future research that includes both quantitative and qualitative methods is needed to identify and address a number of these other potential constraints in accessing MCH services and implementing CCT programs.

## Conclusions

The CCT program had a limited number of positive impacts on MCH service utilization and the knowledge of mothers; it also had almost no effect on child health outcomes. More research needs to be done to create an effective CCT system, and more emphasis on the quality of the health system should be considered in future efforts.

## Endnote

To ensure the accuracy of the information, enumerators also checked children’s birth certificate (place of delivery, birth height, and weight) as well as their vaccination record booklet (vaccinations and physical checkups) on the spot.

## Supplementary information

**Additional file 1: Appendix A1.** English translation of the knowledge test. **Appendix Table A2.** Heterogeneous analysis result by time factor. **Appendix Table A3.** Intention-to-treat (ITT) analysis for the ethnicity and education effects on using MCH services, mother’s knowledge, and child health outcomes. **Appendix Table A4.** Details of the regression specification for Table 3.

## Data Availability

The datasets generated during and/or analyzed during the current study are not publicly available but are available from the corresponding author upon reasonable request.

## Abbreviations

CCT: Conditional Cash Transfer
MCH: Maternal and Child Health
UNICEF: United Nations International Children’s Emergency Fund
ITT: Intention-to-treat
ATT: Average-treatment-effects-on-the-treated
IE: Ineligible for the CCT program
FE: Fully eligible for the CCT program
PE: Partially eligible for the CCT program
SD: Standard deviation
LAZ: Length-for-age *Z*-scores
WLZ: Weight-for-length *Z*-scores

## References

[1] World Health Organization (2016). Sustainable Development Goals (SDGs).

[2] Liao N (2009). The effect of reducing maternal mortality program and NCMS on increasing rate of delivery in hospital. Chin Prim Health Care.

[3] Luo S, An N (2010). Trend analysis on China institutional delivery and causes of maternal death change in 1999-2008. Chin J Woman Child Health.

[4] Liang J, Zhu J, Wang Y (2007). Analysis on factors affecting maternal mortality in China. Chin J Epidemiol.

[5] Minister of Health (2009). The 4th National Health Services Survey. Chin Commun Health.

[6] Wu F, Cao J, Xiong Q (2010). Investigation on the rate of hospital delivery and strategy research in minority concentrated region in Sichuan. Chin Mater Child Care.

[7] Wu Y, Hao G, Sun S, Chen Y, Zhang R, Liu Q (2015). Research on maternal health behaviors for Yi-nationality women in poor rural areas based on the theory of reasoned action. Chin J Prev Med.

[8] Gao Y, Zhou H, Singh NS (2017). Progress and challenges in maternal health in western China: a countdown to 2015 national case study. Lancet Glob Health.

[9] He C, Liu L, Chu Y (2017). National and subnational all-cause and cause-specific child mortality in China, 1996–2015: a systematic analysis with implications for the sustainable development goals. Lancet Glob Health.

[10] Wang T, Li X, Wu Z (2005). Determinants of hospital delivery in rural China: a multiple logistic regression analysis. Chin Prim Health Care.

[11] Yu C, Li X (2008). Two-level logistic modeling analysis on the factors that influence birth in hospitals in poor rural areas of Sichuan province. J Sichuan Univ.

[12] Wang Q, Guo G, Xie W (2013). Influencing factors of hospital delivery in remote poverty mountain areas. Chin J Public Health.

[13] Cui Y, Yang L, Wu Q, Tian X, Zhao Y, Xu Y (2008). A study on influencing factors of hospital delivery of rural pregnant women in Tibet. Chin J Woman Child Health Res.

[14] Rong Y, Sun J (2011). Analysis on the impact factors of hospital delivery in rural ethnic minorities areas. Med Soc.

[15] Liu C, Zhang L, Shi Y (2016). Maternal health services in China’s western rural areas: uptake and correlates. Chin Agri Econ Rev.

[16] Ariel F, Norbert S, Francisco H, Ferreira G (2009). Conditional cash transfers: reducing present and future poverty.

[17] Bourguignon F, Ferreira FHG, Leite PG (2003). Conditional cash transfers, schooling, and child labor: micro-simulating Brazil's Bolsa Escola program. World Bank Econ Rev.

[18] Engle PL, Fernald LC, Alderman H, Behrman J, O'Gara C, Yousafzai A (2011). Strategies for reducing inequalities and improving developmental outcomes for young children in low-income and middle-income countries. Lancet.

[19] Fernald LC, Gertler PJ, Neufeld LM (2008). Role of cash in conditional cash transfer programmes for child health, growth, and development: an analysis of Mexico's Oportunidades. Lancet.

[20] Glassman A (2013). Impact of conditional cash transfers on maternal and newborn health. J Health Popul Nutr.

[21] Owusu-Addo E, Renzaho AMN, Smith BJ (2018). The impact of cash transfers on social determinants of health and health inequalities in sub-Saharan Africa: a systematic review. Health Policy Plan.

[22] Owusu-Addo E, Cross R (2014). The impact of conditional cash transfers on child health in low-and middle-income countries: a systematic review. Int J Public Health.

[23] Moore C (2008). Assessing Honduras’ CCT programme PRAF (Programa de Asignacion familiar): expected and unexpected realities.

[24] Cohen J, Rothschild C, Golub G, Omondi G, Kruk M, McConnell M (2017). Measuring The Impact Of Cash Transfers And Behavioral ‘Nudges’ On Maternity Care In Nairobi, Kenya. Health Aff (Millwood).

[25] Mo D, Zhang L, Yi H (2013). School dropouts and conditional cash transfers: evidence from a randomised controlled trial in rural China's junior high schools. J Dev Stud.

[26] Yefu Z, Meimei J, Jiaojiao Z (2018). Effect of a conditional cash transfer program on nutritional knowledge and food practices among caregivers of 3–5-year-old left-behind children in the rural Hunan Province. Int J Environ Res Public Health.

[27] Yip R, Parvanta I, Cogswell ME (1998). Recommendations to prevent and control iron deficiency in the United States. MMWR.

[28] Mercedes O, Monika B (1997). WHO global database on child growth and malnutrition.

[29] Pei S, Chu K, Evropi T, Neneh R, Xue L, Lin A (2016). Barriers to hospital deliveries among ethnic minority women with religious beliefs in China: a descriptive study using interviews and survey data. Int J Environ Res Public Health.

[30] Zhou H, Sun S, Luo R, Sylvia S, Yue A, Shi Y (2016). Impact of text message reminders on caregivers’ adherence to a home fortification program against child anemia in rural Western China: a cluster-randomized controlled trial. Am J Public Health.

[31] Equator Network (2019). The Strengthening the Reporting of Observational Studies in Epidemiology (STROBE) statement: guidelines for reporting observational studies.

[32] Rivera JA, Sotresalvarez D, Habicht JP, Shamah T, Villalpando S (2004). Impact of the Mexican program for education, health, and nutrition (Progresa) on rates of growth and Anemia in infants and young children: a randomized effectiveness study. JAMA.

[33] Shei A (2013). Brazil’s conditional cash transfer program associated with declines in infant mortality rates. Health Aff (Millwood).

[34] Maluccio J, Flores R (2005). Impact evaluation of a conditional cash transfer program: the Nicaraguan red de Protección social. Res Repo.

[35] Paxson C, Schady N. Does money matter? The effects of cash transfers on child health and development in rural Ecuador: unpublished manuscript: Princeton University and The World Bank; 2007. https://ssrn.com/abstract=984618. Accessed 11 Oct 2018.

[36] Attanasio O, Battistin E, Fitzsimons E, Mesnard A, Vera-Hernandez M (2005). How effective are conditional cash transfers? Evidence from Colombia.

[37] Harris A, Zhou Y, Liao H, Barclay L, Zeng W, Gao Y (2010). Challenges to maternal health care utilization among ethnic minority women in a resource-poor region of Sichuan Province, China. Health Policy Plan.

[38] Adams V, Miller S, Chertow J, Sienna Craig S, Samen A, Varner M (2005). Having a "safe delivery": conflicting views from Tibet. Health Care Women Int.

[39] Huang Y, David S, Pi L, Tian F, Pan J, Ronsmans C. Ethnicity and maternal and child health outcomes and service coverage in western China: a systematic review and meta-analysis. Lancet Glob Health. 2017;6(1):e39–e56. 10.1016/S2214-109X(17)30445-X 29153766

[40] Barber SL, Gertler PJ (2009). Empowering women to obtain high quality care: evidence from an evaluation of Mexico's conditional cash transfer programme. Health Policy Plan.

[41] Wang Y, Zhang J (2012). Qualitative research of rural maternal and child health related behaviour in Liangshan Yi autonomous prefecture in Sichuan Province. Sichuan Med J.

[42] Wen C, Sun J, Yao L (2011). Investigation of maternal care utilization rate for women in minority areas. Chin J Hosp Adm.

[43] Sylvia S, Shi Y, Xue H, Tian X, Wang H, Liu Q (2015). Survey using incognito standardized patients shows poor quality care in China's rural clinics. Health Policy Plan.

[44] Sylvia S, Xue H, Zhou C, Shi Y, Yi H, Zhou H (2017). Tuberculosis detection and the challenges of integrated care in rural China: a cross- sectional standardized patient study. PLoS Med.

[45] Li X, Lu S, Hu S, Cheng K, Maeseneer JD, Meng Q (2017). The primary health-care system in China. Lancet.

