# Supplementary material for: Conditional cash transfers, uptake of maternal and child health services, and health outcomes in western rural China
Source: BMC Public Health. 2020 Jun 5;20:870. doi: 10.1186/s12889-020-08996-9 (PMC7275386; doi:10.1186/s12889-020-08996-9)
Supplement: Supplementary file 1 — Additional file 1: Appendix A1. English translation of the knowledge test. Appendix Table A2. Heterogeneous analysis result by time factor. Appendix Table A3. Intention-to-treat (ITT) analysis for the ethnicity and education effects on using MCH services, mother’s knowledge, and child health outcomes. Appendix Table A4. Details of the regression specification for Table 3. [file 12889_2020_8996_MOESM1_ESM.docx]

**Additional File**

Supplement to:

**Conditional cash transfers, uptake of maternal and child health services, and health outcomes in western rural China**

**Table of Contents**

Appendix A1: English translation of the knowledge test

Appendix Table A2: Heterogeneous analysis result by time factor

Appendix Table A3: Intention-to-treat (ITT) analysis for the ethnicity and education effects on using MCH services, mother’s knowledge, and child health outcomes

Appendix Table A4: Details of the regression specification for Table 3

**Appendix A1**

English translation of the knowledge test

1. Do you think that smoking can cause an abortion?

1 = Yes, 2 = No, 3 = Don’t know

1. Do you think that the close contact of pregnant women with cats, dogs, and other animals will have a negative impact on the baby?

1 = Yes, 2 = No, 3 = Don’t know

1. Do you think that pregnant women should see a doctor if they encounter vaginal bleeding?

1 = Yes, 2 = No, 3 = Don’t know

1. Do you think that pregnant women should see a doctor if they experience abdominal pain?

1 = Yes, 2 = No, 3 = Don’t know

1. Do you think that pregnant women should see a doctor if they rupture a membrane?

1 = Yes, 2 = No, 3 = Don’t know

1. Do you think that pregnant women should see a doctor if they experience swelling of the lower limbs?

1 = Yes, 2 = No, 3 = Don’t know

1. Do you think that pregnant women with abnormal fetal movement should see a doctor?

1 = Yes, 2 = No, 3 = Don’t know

1. At least how many antenatal examinations do you think that pregnant women should undertake? _________
2. Do you think women should take folic acid during pregnancy?

1 = Yes, 2 = No, 3 = Don’t know

1. Do you think women should take vitamins during pregnancy?

1 = Yes, 2 = No, 3 = Don’t know

1. Do you think women should take calcium during pregnancy?

1 = Yes, 2 = No, 3 = Don’t know

1. Do you think women should take iron during pregnancy?

1 = Yes, 2 = No, 3 = Don’t know

1. Do you think the mother and her baby should have a health checkup after delivery?

1 = Yes, 2 = No, 3 = Don’t know

1. How many days after childbirth do you think the mother and her baby should go before a health checkup?
2. What do you think the birth weight for a normal-term newborn baby is?

1 = Less than 2.5 kilograms, 2 = 2.5–4 kilograms, 3 = More than 4 kilograms,

4 = Don’t know

1. When do you think a baby’s deciduous teeth erupt?

1 = Within 3 months, 2 = 4–10 months, 3 = 10–15 months,

4 = More than 15 months

1. Do you think a baby needs vitamin D from birth?

1 = Yes, 2 = No, 3 = Don’t know

1. Do you think full-term infants need calcium supplementation six months after birth?

1 = Yes, 2 = No, 3 = Don’t know

1. What do you think is the best time to begin to breastfeed a baby?

1 = Within 1 hour after delivery, 2 = 2–12 hours after delivery,

3 = 13–24 hours after delivery, 4 = More than 24 hours after delivery

1. For at least how long do you think exclusive breastfeeding should last? _________ months
2. When do you think is the best time to start giving supplements to a baby? by_________ months
3. How many health checkups should a baby have taken by the age of 12 months? _________.

**Appendix** **Table A2**

Heterogeneous analysis result by time factor (*N* = 1,522)

| **Dependent variable** | CCT | |  | Time ^a^ | |  | CCT*Time ^b^ | |
| --- | --- | --- | --- | --- | --- | --- | --- | --- |
|  | $\boldsymbol{\beta}$ | *p* |  | $\boldsymbol{\beta}$ | *p* |  | $\boldsymbol{\beta}$ | *p* |
|  | (1) | (2) |  | (3) | (4) |  | (5) | (6) |
| ***Uptake of MCH services*** |  |  |  |  |  |  |  |  |
| (1) Any antenatal examination (%) | 0.25 | 0.44 |  | -0.62 | 0.13 |  | 0.63 | 0.24 |
| (2) Hospital delivery (%) | 0.22 | 0.53 |  | -0.92 | 0.00 |  | 0.82 | 0.07 |
| (3) Postpartum visits (%) | 0.45 | 0.03 |  | -0.63 | 0.02 |  | 0.68 | 0.07 |
| (4) Early breastfeeding (%) | 0.22 | 0.30 |  | 0.45 | 0.07 |  | -0.62 | 0.09 |
| (5) Exclusive breastfeeding (%) | 0.32 | 0.07 |  | 0.10 | 0.61 |  | -0.38 | 0.41 |
| (6) Compliance rate of physical examination, (%)^c^ | 0.53 | 0.10 |  | -0.75 | 0.07 |  | 1.19 | 0.08 |
| (7) Compliance rate of child vaccinations, (%)^c^ | 0.33 | 0.27 |  | -0.38 | 0.20 |  | 0.82 | 0.05 |
| ***Mother’s knowledge*** |  |  |  |  |  |  |  |  |
| (8) Total knowledge scores (full = 22) | 0.84 | 0.04 |  | -0.40 | 0.30 |  | 0.97 | 0.23 |
| (9) Got at least 60% correct (%) | 0.39 | 0.07 |  | 0.18 | 0.54 |  | 0.02 | 0.97 |
| (10) Score on maternal care (full = 8) | 0.32 | 0.04 |  | -0.30 | 0.14 |  | 0.43 | 0.26 |
| (11) Score on child nutrition (full = 6) | 0.24 | 0.04 |  | -0.08 | 0.58 |  | 0.24 | 0.31 |
| (12) Thinking child physical examination necessary (%) | 0.55 | 0.05 |  | -0.15 | 0.65 |  | 0.52 | 0.33 |
| ***Child health outcomes*** |  |  |  |  |  |  |  |  |
| (13) Low birth weight (%) | -0.23 | 0.48 |  | -0.27 | 0.49 |  | 1.26 | 0.03 |
| (14) Anemia (%) | -0.14 | 0.48 |  | 0.16 | 0.48 |  | 0.45 | 0.34 |
| (15) Growth retardation (%)^d^ | -0.09 | 0.65 |  | -0.13 | 0.69 |  | -0.60 | 0.29 |
| (16) Stunting (%)^e^ | -0.04 | 0.94 |  | 0.67 | 0.14 |  | -0.91 | 0.46 |

The results present the interaction effect of CCT and time on uptake of health services, mother’s knowledge, and child health outcomes.

Covariates include child’s age, gender, low birth weight, premature birth, and birth order; and mother’s occupation, number of children, whether the family received social security support, and household fixed assets. Standard errors are clustered at the town level.

^a^: Travel time from home to township center: 1 = more than 1 hour, 0 = 1 or less than 1 hour.

^b^: Women spent more than 1 hour on traveling from home to the township health center and accepted CCT program.

^c^: Compliance rates of physical examinations and child vaccinations are calculated by the requirements of the national standards of basic public health services by child’s age.

^d^: Stunted growth: length-for-age *Z*-scores are less than -2 standard deviations.

^e^: Wasting: weight-for-height *Z*-scores are less than -2 standard deviations.

*Source: authors’ survey.*

**Appendix Table A3**

Intention-to-treat (ITT) analysis for the ethnicity and education effects (*N* = 1,522)

| **Dependent variable** | **FE-CCT Versus**  **FE-comparison** | |  | **PE-CCT Versus**  **PE-comparison** | |  | **FE-CCT Versus**  **IE-CCT** | |  | **PE-CCT Versus**  **IE-CCT** | |
| --- | --- | --- | --- | --- | --- | --- | --- | --- | --- | --- | --- |
|  | **Ethnicity** | **Education** |  | **Ethnicity** | **Education** |  | **Ethnicity** | **Education** |  | **Ethnicity** | **Education** |
|  | (1) | (2) |  | (3) | (4) |  | (5) | (6) |  | (7) | (8) |
| ***Uptake of MCH services*** |  |  |  |  |  |  |  |  |  |  |  |
| (1) Any antenatal examination (%) | -1.47* | 1.76* |  | -2.19* | 2.21* |  | -1.64* | 2.58* |  | -1.38* | 0.00 |
| (2) Hospital delivery (%) | -2.41* | 1.15* |  | -2.30* | 1.41* |  | -2.34* | 0.86 |  | -2.14* | 1.22* |
| (3) Postpartum visits (%) | -1.24* | 0.90* |  | -1.86* | 0.77* |  | -1.45* | 1.05* |  | -1.51* | 1.38* |
| (4) Early breastfeeding (%) | 0.66* | -0.16 |  | 0.08 | -0.54 |  | 0.13 | 0.03 |  | 0.15 | -0.16 |
| (5) Exclusive breastfeeding (%) | -0.66* | 0.77* |  | -0.75* | 0.31 |  | -0.59 | 0.37 |  | -1.16* | -0.12 |
| (6) Compliance rate of physical examination, (%)^a^ | 0.04 | 0.38 |  | 0.37 | 1.24* |  | 0.04 | 0.23 |  | 0.00 | 0.08 |
| (7) Compliance rate of child vaccinations, (%)^a^ | -0.71* | 0.38 |  | -0.34 | 0.80* |  | -1.54* | 1.11* |  | -1.32* | 0.55 |
| ***Mother’s knowledge*** |  |  |  |  |  |  |  |  |  |  |  |
| (8) Total knowledge scores (full = 22) | -1.98* | 1.83* |  | -2.41* | 2.54* |  | -0.77 | 3.25* |  | -1.78* | 3.44* |
| (9) Got at least 60% correct (%) | -0.72* | 0.74* |  | -1.10* | 1.49* |  | -0.07 | 1.48* |  | -0.80* | 1.87* |
| (10) Score on maternal care (full = 8) | -0.65* | 0.59* |  | -0.66* | 0.95* |  | -0.33 | 1.01* |  | -0.60* | 1.11* |
| (11) Score on child nutrition (full = 6) | -0.56* | 0.43* |  | -0.78* | 0.35* |  | -0.28 | 0.79* |  | -0.51* | 0.73* |
| (12) Thinking child physical examination necessary (%) | -0.51 | 0.36 |  | -0.54 | 1.08* |  | -0.33 | 1.08* |  | -0.60 | 1.08* |
| ***Child health outcomes*** |  |  |  |  |  |  |  |  |  |  |  |
| (13) Low birth weight (%) | 0.08 | -0.63 |  | -0.17 | -0.63 |  | 0.52 | -0.77 |  | -0.88 | -0.54 |
| (14) Anemia (%) | 0.20 | -0.38 |  | -0.71* | -0.37 |  | 0.52 | -0.04 |  | -0.50 | 0.00 |
| (15) Growth retardation (%)^b^ | 0.19 | -0.53 |  | -0.21 | -0.13 |  | 1.50* | 0.09 |  | 0.55 | 0.07 |
| (16) Stunting (%)^c^ | -0.33 | 0.16 |  | 0.73 | -0.44 |  | -1.05 | 0.46 |  | 0.96 | 0.78 |

The linear and logistic regression are used to analyze the influence of ethnicity and education on uptake of health services, mother’s knowledge, and child health outcomes. Covariates include child’s age, gender, low birth weight, premature birth, and birth order; and mother’s occupation, number of children, whether the family received social security support, distance from household to township heath center, travel time from household to township health center, and household fixed assets. Standard errors are clustered at the town level. FE = fully eligible for the CCT program; PE = partially eligible; IE =ineligible.

^a^: Compliance rates of physical examinations and child vaccinations are calculated by the requirements of the national standards of basic public health services by child’s age.

^b^: Stunted growth: length-for-age *Z*-scores are less than -2 standard deviations.

^c^: Wasting: weight-for-height *Z*-scores are less than -2 standard deviations.

**p* < 0.05

*Source: authors’ survey.*

**Appendix Table A4**

A4.1 Details of the regression specification for Table 3: FE-CCT vs. FE comparison

| **Regression** | **Samples** | **Independent variable** | **Dependent variables** | **Coefficients** | **Standard errors** | ***F* value/**  **Wald** $\boldsymbol{\chi}^{\boldsymbol{2}}$ | ***R^2^*** |
| --- | --- | --- | --- | --- | --- | --- | --- |
| (1) | 527 | Any antenatal examination (%) | ***Child characteristics*** | 0.44 | 0.38 | 41.93 | 0.18 |
| (2) | 527 | Hospital delivery | Age, (months) | 0.01 | 0.37 | 61.07 | 0.24 |
| (3) | 527 | Postpartum visits | Gender, (%) | 0.23 | 0.27 | 96.87 | 0.23 |
| (4) | 527 | Early breastfeeding | First pregnancy, (%) | 0.02 | 0.25 | 46.43 | 0.10 |
| (5) | 296 | Exclusive breastfeeding | ***Mother characteristics*** | 0.55 | 0.32 | 37.19 | 0.12 |
| (6) | 527 | Compliance rate of physical examination | Age, (years) | 0.10 | 0.05 | 3.23 | 0.08 |
| (7) | 527 | Compliance rate of child vaccinations | Non-Han ethnicity,( %) | 0.04 | 0.04 | 5.06 | 0.11 |
| (8) | 527 | Total knowledge scores | Junior high school and above education, (%) | 0.91 | 0.55 | 18.39 | 0.30 |
| (9) | 527 | Got at least 60% correct | Farmer or housewife, (%) | 0.58 | 0.29 | 82.25 | 0.13 |
| (10) | 527 | Score on maternal care | ***Household characteristics*** | 0.15 | 0.25 | 7.24 | 0.18 |
| (11) | 527 | Score on child nutrition | Number of children aged 0-5 years old | 0.39 | 0.16 | 7.89 | 0.16 |
| (12) | 527 | Thinking child physical examination necessary | Travel time from household to THC^a^ more than 1 hour,(%) | 0.66 | 0.37 | 32.05 | 0.07 |
| (13) | 441 | Low birth weight | Subsistence allowance recipients, (%) | -0.40 | 0.47 | 14.38 | 0.08 |
| (14) | 405 | Anemia | Households ranked the poorest quartile in terms of fixed assets^b^, (%) | -0.19 | 0.25 | 20.89 | 0.04 |
| (15) | 456 | Stunted growth |  | 0.19 | 0.35 | 24.15 | 0.10 |
| (16) | 494 | Wasting |  | 0.13 | 0.15 | 30.75 | 0.06 |

Baby age and gender are not included in dependent variables of regressions (1) and (2).

*Source: authors’ survey.*

A4.2 Details of the regression specification for Table 3: PE-CCT vs. PE comparison

| **Regression** | **Samples** | **Independent variable** | **Dependent variables** | **Coefficients** | **Standard errors** | ***F* value/**  **Wald** $\boldsymbol{\chi}^{\boldsymbol{2}}$ | ***R^2^*** |
| --- | --- | --- | --- | --- | --- | --- | --- |
| (1) | 514 | Any antenatal examination (%) | ***Child characteristics*** | 0.41 | 0.35 | 61.36 | 0.21 |
| (2) | 514 | Hospital delivery | Age, (months) | 0.38 | 0.37 | 67.05 | 0.27 |
| (3) | 514 | Postpartum visits | Gender, (%) | 0.84 | 0.29 | 103.45 | 0.24 |
| (4) | 514 | Early breastfeeding | First pregnancy, (%) | 0.06 | 0.25 | 68.53 | 0.11 |
| (5) | 511 | Exclusive breastfeeding | ***Mother characteristics*** | 0.05 | 0.25 | 19.24 | 0.04 |
| (6) | 514 | Compliance rate of physical examination | Age, (years) | 0.13 | 0.06 | 5.78 | 0.12 |
| (7) | 514 | Compliance rate of child vaccinations | Non-Han ethnicity,( %) | 0.02 | 0.04 | 11.07 | 0.18 |
| (8) | 514 | Total knowledge scores | Junior high school and above education, (%) | 0.82 | 0.45 | 24.90 | 0.33 |
| (9) | 514 | Got at least 60% correct | Farmer or housewife, (%) | 0.30 | 0.28 | 61.45 | 0.14 |
| (10) | 514 | Score on maternal care | ***Household characteristics*** | 0.48 | 0.20 | 11.40 | 0.19 |
| (11) | 514 | Score on child nutrition | Number of children aged 0-5 years old | 0.22 | 0.13 | 6.74 | 0.14 |
| (12) | 514 | Thinking child physical examination necessary | Travel time from household to THC^a^ more than 1 hour,(%) | 0.87 | 0.34 | 85.50 | 0.15 |
| (13) | 434 | Low birth weight | Subsistence allowance recipients, (%) | 0.23 | 0.39 | 84.59 | 0.16 |
| (14) | 400 | Anemia | Households ranked the poorest quartile in terms of fixed assets^b^, (%) | 0.27 | 0.25 | 23.66 | 0.06 |
| (15) | 478 | Stunted growth |  | -0.42 | 0.40 | 45.98 | 0.13 |
| (16) | 477 | Wasting |  | 0.36 | 0.21 | 10.86 | 0.04 |

Baby age and gender are not included in dependent variables of regressions (1) and (2).

*Source: authors’ survey.*

A4.3 Details of the regression specification for Table 3: FE-CCT vs. IE-CCT

| **Regression** | **Samples** | **Independent variable** | **Dependent variables** | **Coefficients** | **Standard errors** | ***F* value/**  **Wald** $\boldsymbol{\chi}^{\boldsymbol{2}}$ | ***R^2^*** |
| --- | --- | --- | --- | --- | --- | --- | --- |
| (1) | 328 | Any antenatal examination (%) | ***Child characteristics*** | 0.14 | 0.21 | 100.19 | 0.21 |
| (2) | 328 | Hospital delivery | Age, (months) | 0.10 | 0.27 | 29.97 | 0.23 |
| (3) | 328 | Postpartum visits | Gender, (%) | 0.28 | 0.72 | 86.28 | 0.22 |
| (4) | 328 | Early breastfeeding | First pregnancy, (%) | -1.07 | 0.42 | 32.37 | 0.04 |
| (5) | 252 | Exclusive breastfeeding | ***Mother characteristics*** | -0.06 | 0.87 | 33.32 | 0.07 |
| (6) | 328 | Compliance rate of physical examination | Age, (years) | 0.10 | 0.04 | 3.41 | 0.06 |
| (7) | 328 | Compliance rate of child vaccinations | Non-Han ethnicity,( %) | 0.01 | 0.05 | 20.94 | 0.20 |
| (8) | 328 | Total knowledge scores | Junior high school and above education, (%) | 0.33 | 0.89 | 12.27 | 0.30 |
| (9) | 328 | Got at least 60% correct | Farmer or housewife, (%) | 0.66 | 0.53 | 61.45 | 0.14 |
| (10) | 328 | Score on maternal care | ***Household characteristics*** | 0.51 | 0.49 | 9.83 | 0.16 |
| (11) | 328 | Score on child nutrition | Number of children aged 0-5 years old | -0.14 | 0.30 | 6.74 | 0.14 |
| (12) | 328 | Thinking child physical examination necessary | Travel time from household to THC^a^ more than 1 hour,(%) | 1.97 | 1.07 | 85.50 | 0.15 |
| (13) | 231 | Low birth weight | Subsistence allowance recipients, (%) | 1.03 | 1.37 | 84.96 | 0.16 |
| (14) | 259 | Anemia | Households ranked the poorest quartile in terms of fixed assets^b^, (%) | 0.00 | 0.58 | 23.66 | 0.06 |
| (15) | 306 | Stunted growth |  | 0.23 | 0.71 | 45.98 | 0.13 |
| (16) | 305 | Wasting |  | -0.73 | 0.41 | 10.86 | 0.04 |

Baby age and gender are not included in dependent variables of regressions (1) and (2).

*Source: authors’ survey.*

A4.4 Details of the regression specification for Table 3: PE-CCT vs. IE-CCT

| **Regression** | **Samples** | **Independent variable** | **Dependent variables** | **Coefficients** | **Standard errors** | ***F* value/**  **Wald** $\boldsymbol{\chi}^{\boldsymbol{2}}$ | ***R^2^*** |
| --- | --- | --- | --- | --- | --- | --- | --- |
| (1) | 329 | Any antenatal examination (%) | ***Child characteristics*** | -0.10 | 0.32 | 22.67 | 0.12 |
| (2) | 329 | Hospital delivery | Age, (months) | 0.26 | 0.31 | 22.65 | 0.24 |
| (3) | 329 | Postpartum visits | Gender, (%) | 0.27 | 0.36 | 111.48 | 0.20 |
| (4) | 329 | Early breastfeeding | First pregnancy, (%) | -0.51 | 0.29 | 46.43 | 0.05 |
| (5) | 324 | Exclusive breastfeeding | ***Mother characteristics*** | -0.29 | 0.52 | 38.81 | 0.08 |
| (6) | 329 | Compliance rate of physical examination | Age, (years) | -0.05 | 0.04 | 1.93 | 0.06 |
| (7) | 329 | Compliance rate of child vaccinations | Non-Han ethnicity,( %) | -0.05 | 0.04 | 8.42 | 0.24 |
| (8) | 329 | Total knowledge scores | Junior high school and above education, (%) | -0.29 | 0.06 | 29.29 | 0.34 |
| (9) | 329 | Got at least 60% correct | Farmer or housewife, (%) | 0.06 | 0.33 | 109.01 | 0.22 |
| (10) | 329 | Score on maternal care | ***Household characteristics*** | 0.28 | 0.29 | 11.61 | 0.16 |
| (11) | 329 | Score on child nutrition | Number of children aged 0-5 years old | -0.17 | 0.17 | 5.40 | 0.18 |
| (12) | 329 | Thinking child physical examination necessary | Travel time from household to THC^a^ more than 1 hour,(%) | 1.40 | 0.71 | 87.58 | 0.15 |
| (13) | 273 | Low birth weight | Subsistence allowance recipients, (%) | 0.68 | 0.69 | 54.08 | 0.13 |
| (14) | 265 | Anemia | Households ranked the poorest quartile in terms of fixed assets^b^, (%) | 0.91 | 0.33 | 55.30 | 0.14 |
| (15) | 310 | Stunted growth |  | -0.58 | 0.56 | 46.93 | 0.09 |
| (16) | 308 | Wasting |  | 1.15 | 0.34 | 12.86 | 0.05 |

*Source: authors’ survey.*
